# Relationship between the AB0 Blood System and Proximal Femoral Fracture Patterns in the Turkish Population

**DOI:** 10.1155/2020/1834525

**Published:** 2020-03-20

**Authors:** Tolgahan Kuru, Haci Ali Olcar

**Affiliations:** ^1^Department of Orthopedics and Traumatology, Onsekiz Mart University Medical Faculty, Canakkale, Turkey; ^2^Department of Orthopedics and Traumatology, Yozgat Public Hospital, Turkey

## Abstract

**Objective:**

AB0 blood groups have shown to be associated with increased risk of several orthopedic disorders such as Achilles tendon rupture and hip osteoarthritis. The objective of this study was to investigate relationships between the AB0 blood system and hip fracture patterns, duration of hospitalization, and amount of blood transfusion.

**Methods:**

Data of 308 patients treated due to hip fractures in our hospital between 2017 and 2019 were evaluated. Epicrisis reports and X-rays of the patients included in this study were retrospectively screened, and hip fractures were classified as intracapsular and extracapsular fractures. Patients were divided into A, B, 0, and AB groups according to blood groups.

**Results:**

The mean age of the patients was 75.54 ± 13.5 years. Of all patients, 103 had an intracapsular fracture and 205 had an extracapsular fracture. No statistically significant correlation was found between blood groups and fracture patterns. No statistically significant difference was found between the amounts of transfusion according to the blood groups, and no significant difference between the durations of hospitalizations according to the blood groups.

**Conclusion:**

In our study population, we could not find a significant relationship between the AB0 blood system and fracture patterns. We think that these potential relationships could be explained with further comprehensive studies with larger populations.

## 1. Introduction

Hip fractures remain the most important fracture because of poor survival and requiring immediate surgery. Proximal femoral fractures account for a significant part of hospitalizations among trauma cases. The overall number of proximal femoral fractures is constantly increasing worldwide [1]. Hip fractures constitute 20% of the workload in orthopedic trauma units [2]. Hip fractures are an important cause of morbidity and mortality, resulting in increasing healthcare costs. In general, hip fractures are thought to be a single entity. However, intracapsular and extracapsular fractures have different bone composition, proximal femoral geometric parameters, epidemiology, and clinical characteristics [3, 4]. In their study, Tanner et al. found that advanced age was associated with increasing incidence of intertrochanteric (extracapsular) fractures in women and increasing incidence of intracapsular fractures in men [5]. Mechanisms leading to fracture patterns have not yet been fully understood. Age, parathyroid hormone, vitamin D, distribution of bone mass density, proximal femoral geometry, lifestyle, and nutritional status are the possible related causes [6, 7]. Fisher et al. found vitamin D insufficiency in most of the patients with hip fracture [6]. People with increased parathyroid hormone level in response to vitamin D insufficiency tend to have extracapsular fractures, while persons with muted parathyroid hormone response presented more commonly due to intracapsular fractures.

Thorsoe underlined the possible role of AB0 blood groups in the 1960s [8]. Several studies have associated blood groups with ageing [9]. In recent years, AB0 antigens have been associated with cerebrovascular and cardiovascular diseases, visceral neoplasia, and infections [10]. In addition, AB0 blood groups have shown to be associated with increased risk of venous thromboembolic and arterial disease [4]. Nevertheless, the current epidemiological data could not provide a coherent picture of the relationship between AB0 blood groups and thromboembolic risk. However, there are studies reporting an association between blood groups and diseases such as Achilles tendon rupture [11] and hip osteoarthritis [12]. The association between the distribution of AB0 blood groups and osteoporosis has been confirmed [13]. These findings suggest that the gene and enzymes in the AB0 system have pleiotropic roles. Blood groups show a different distribution all over the world.

The objective of this study was to investigate relationships between the AB0 blood system and hip fracture patterns, duration of hospitalization, and amount of blood transfusion.

## 2. Material and Methods

Patients operated in our tertiary application and research hospital, which has a capacity of 565 beds and 96 intensive care beds, between 2017 and 2019 were retrospectively screened from the MIA-MED hospital information management system (MIA Teknoloji, Ankara, Turkey) using OCD and SUT codes. Epicrisis and X-rays of the patients included in this study were retrospectively screened, and hip fractures were classified as intracapsular and extracapsular fractures. Patients were divided into A, B, 0, and AB groups according to blood groups, in order to determine whether blood pressure was associated with fracture patterns, amount of transfusion, and duration of hospitalization. Information on AB0 blood groups was obtained from records of the hematology unit. A univariate logistic regression analysis was performed to determine the factors affecting the amount of blood transfusion and hospitalization. Since no statistical significance was found, no multivariate analysis was performed.

Patients' demographic data such as age and gender, preoperative blood groups, fracture patterns, surgical procedure performed, amount of blood transfusion, and duration of hospitalization were recorded. Based on the retrospective data screening, patients with a missing blood group pattern, patients whose preop X-ray was not available, those with missing data in the system about the amount of transfusion, and patients with a hip fracture other than the intra- or extracapsular fracture were excluded from the study. The study was approved by the local ethics committee of Yozgat Bozok University (date 29/05/2019 and decision number 2017-KAEK-189-2019.05.29_20) and was conducted in line with the principles of the Declaration of Helsinki.

### 2.1. Statistical Analysis

Data were analyzed using IBM Statistical Package for the Social Sciences (SPSS) version 22 statistical software (SPSS Inc., Chicago, IL, USA). Normality of the variables was tested with the Kolmogorov-Smirnov test. Nonparametric tests were used for the nonnormally distributed variables. Categorical data were compared with the chi-square test. Normally distributed continuous variables were analyzed with the *t*-test and nonnormally distributed continuous variables with the Mann-Whitney *U* test. Quantitative data are expressed as mean, standard deviation, median, quartile scale, and minimum and maximum values. Confidence interval was taken as 95%. *p* < 0.05 values were considered statistically significant.

## 3. Results

In this study, data of 337 patients treated due to hip fractures in our hospital between 2017 and 2019 were evaluated. A total of 29 patients were excluded from the study after the evaluation of data. Of these patients, 14 were excluded due to missing blood group patterns, 9 because of the lack of preop X-rays, and 6 due to missing data entry for the amount of transfusion. The remaining 308 patients were evaluated retrospectively.

Of all patients, 134 were male and 174 female, with an M/F ratio of 0.77. Fracture patterns and mean duration of hospitalization according to genders are given in Table 1.

The mean age of the patients was 75.54 ± 13.5 years (min–max: 21-97). Fracture patterns and mean duration of hospitalization according to age groups are shown in Table 2.

**Figure 1:**
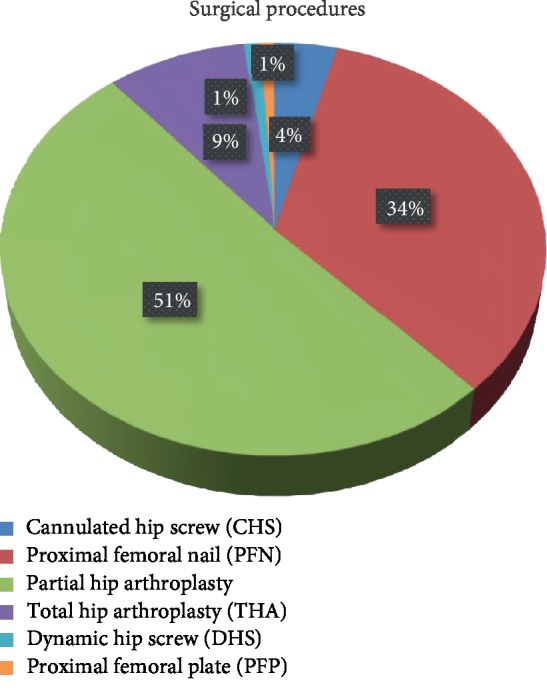
Distribution of the surgical procedures used in patients.

When procedures performed were analyzed, a cannulated screw was used in 12 patients, proximal femoral nail (PFN) was employed in 104 patients, partial hip arthroplasty (PHA) and total hip arthroplasty (THA) were performed in 157 and 27 patients, respectively, a dynamic hip screw (DHS) plate was used in 4 patients, and a proximal femoral plate (PFP) was used in 4 patients (Figure 1).

**Figure 2:**
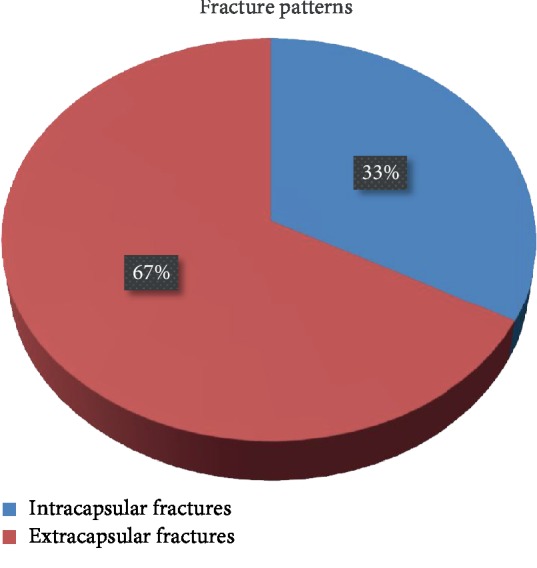
Distribution of fracture patterns.

Of all patients, 103 (33.4%) had an intracapsular fracture and 205 (66.6%) had an extracapsular fracture (Figure 2). The mean age was found to be 72.64 years in patients with an intracapsular fracture and 77.01 years in those with an extracapsular fracture.

**Figure 3:**
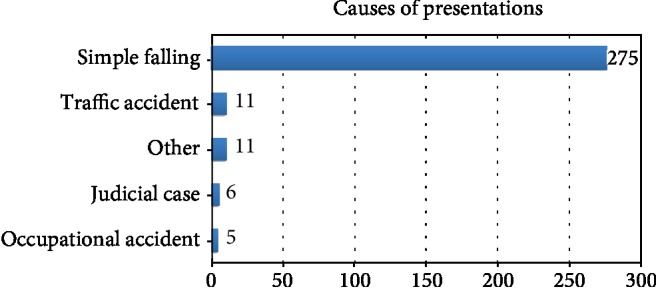
Causes of presentations to the emergency department.

When causes of presentation to the emergency department were examined, 275 patients presented due to trauma (simple falling), 11 patients due to traffic accidents, 5 patients because of occupational accidents, 6 patients due to judicial case, and 11 patients with other reasons (Figure 3).

**Figure 4:**
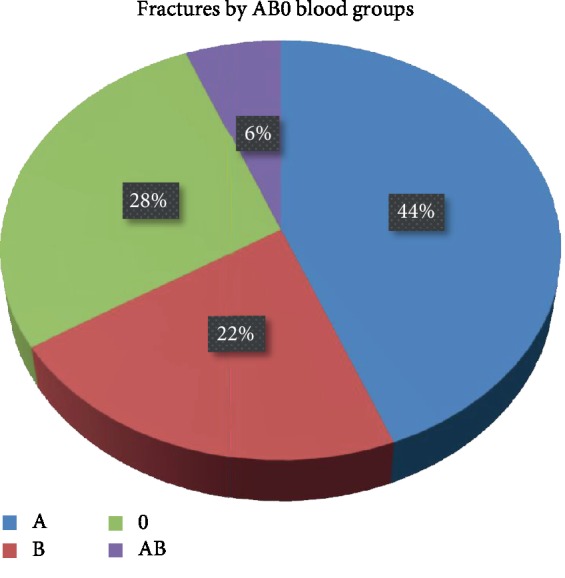
Distribution of fractures by AB0 blood groups.

Overall fracture rates by blood groups were found to be 43.5% (*n* = 134) in group A, 22.4% (*n* = 69) in group B, 27.9% (*n* = 86) in group 0, and 6.2% (*n* = 19) in group AB (Figure 4). The male to female ratio was found to be 0.78 in the A blood group, which was the most common group. Among the patients with intracapsular fractures, blood groups (A, B, 0, and AB) were found in 48 (46.6%), 27 (26.2%), 21 (20.4%), and 7 (6.8%) patients, respectively. Among the patients with extracapsular fractures, blood groups (A, B, 0, and AB) were found in 86 (41.6%), 42 (20.5%), 65 (31.7%), and 12 (5.6%) patients, respectively. No statistically significant correlation was found between blood groups and fracture patterns (*p* = 0.205) (Table 3).

When amounts of transfusion were evaluated by blood groups, the mean transfusion amount was found to be 0.78 U in group A, 0.71 U in group B, 0.9 U in group 0, and 0.63 U in group AB. No statistically significant difference was found between the amounts of transfusion according to the blood groups (*p* = 0.64). Again, there was no significant difference between the durations of hospitalizations according to the blood groups (*p* = 0.206).

The mean duration of hospitalization was found to be 4.78 ± 2.91 days (min–max: 1-27). The mean duration of hospitalization was found to be 4.45 ± 2.47 days (min–max: 1-15) in patients with intracapsular fractures and 4.95 ± 2.9 days (min–max: 1-27) in patients with extracapsular fractures. There was a weak positive correlation between age and duration of hospitalization (*r* = 0.137, *p* = 0.016). However, when the patients were evaluated according to the age groups, the duration of hospitalization was significantly shorter in 46-55 and 56-65 years age groups compared to the >65 years age group. No significant difference was found between genders and duration of hospitalization (*p* = 0.725). There was a significant difference between durations of hospitalization according to the type of the operation performed. Accordingly, the duration of hospitalization was significantly shorter in patients who underwent cannulated screw and PFN operations compared to the other patients (both *p* < 0.05). The mean duration of hospitalization was found to be 2.08 days in patients who underwent the cannulated screw procedure and 4.1 days in patients who underwent PFN. In addition, the mean duration of hospitalization was significantly shorter with the cannulated screw operation, compared to all other surgical procedures (all *p* < 0.05). All patients in the cannulated screw group were in the <66 years age group except for one patient. The mean duration of hospitalization in the PFN group was longer than that in the cannulated screw group and significantly shorter than that in the THA and DHS groups. No significant difference was found between PFN and the other surgical groups in terms of the duration of hospitalization (*p* > 0.05). The mean duration of hospitalization was longer in patients who underwent partial hip arthroplasty (PHA) compared to patients who underwent PFN, although the difference was not statistically significant (*p* > 0.05). The majority of the patients with PFN performed were in the >75 years age group (Table 4).

The mean duration of hospitalization was found to be 4.45 ± 2.47 days (min–max: 1-15) in patients with intracapsular fractures and 4.95 ± 2.9 days (min–max: 1-27) in patients with extracapsular fractures. No statistically significant difference was found between the durations of hospitalization according to fracture patterns (*p* > 0.05).

The overall mean amount of blood transfusion was found to be 0.79 ± 1.28 U in all patients. The amounts of transfusion were found to be 0.72 U for intracapsular and 0.82 U for extracapsular fractures. Accordingly, there was no statistically significant difference between the amounts of blood transfusions according to fracture patterns (*p* = 0.302).

No transfusion was made in a total of 186 patients, while blood transfusion was needed in 122 patients before, after, and during the operation. The maximum amount of blood transfusion performed in the patients was 6 U. There was a weak positive correlation between age and transfusion amount (*r* = 0.181). No significant difference was found between the amounts of blood transfusion according to gender (*p* = 0.303). There was a weak positive correlation between the duration of hospitalization and the amount of transfusion (*r* = 0.249) (Table 5).

It was found that no blood transfusion was made in patients who underwent cannulated screw and plate operations, while the amount of transfusion was found to be 1.04 U in DHS, 0.8 U in THA, and 0.5 U in PFN procedures.

There was a significant difference between the transfusion amounts according to the surgical procedures. In the analysis performed to determine the groups causing a significant difference, it was found that there was a significant difference between amounts of transfusion performed in patients who underwent PFN and those underwent PHA, with a higher amount of transfusion being made in the PFN group.

## 4. Discussion

In the present study, no significant correlations were found between blood groups in terms of fracture patterns, amount of transfusion, and duration of hospitalization. On the other hand, hereditary factors have been implicated among the factors affecting fracture patterns, including AB0 genes. The AB0 blood system, which is the most commonly used system in the classification of human blood, is based on the hereditary features determined by the presence or absence of A and B antigens in red blood and tissue cells, saliva, and body fluids.

Recently, the AB0 system has been associated with several diseases such as cardiovascular disease and pancreatic and gastric cancers [9]. It has been reported that the risk of squamous cell carcinoma was reduced by 14% and the risk of basal cell carcinoma by 5% in persons with a non-0 (A, B, and AB) blood group compared to individuals with a 0 blood group [14]. On the other hand, the 0 blood group has been associated with a decreased risk of pancreatic cancer [15].

The basis of the AB0 blood system has been discovered for the first time by Australian physician Landsteiner and Czech psychologist Janski, independently of each other in the first decade of the 20th century. This is the first identified human polymorphism [16].

Relationships between blood groups and various diseases have been studied for a long time. Orthopedics is one of the fields of research on relevance of blood groups. Studies have found increased incidence and severity of osteoporosis in elderly patients with a non-0 blood group, compared to those with a 0 blood group [13, 17]. Choi and Pai found the highest incidence of osteoporosis and the lowest bone mineral density in patients with the AB blood group [13].

In our study, when distribution of blood groups was evaluated, 43.5% had group A, 22.4% group B, 27.9% group 0, and 6.2% group AB blood. In a study conducted by Ergün et al. on 288,469 subjects, which reflected nationwide data, group A was found in 44.62%, group B in 15.45%, group 0 in 32.24%, and group AB in 7.69% [18]. According to the data published by the Turkish Red Crescent (*n* = 5,816,855), nationwide blood groups (A, B, 0, and AB) in Turkey were reported in 42.5%, 15.8%, 33.7%, and 8.0%, respectively [19]. Our results are consistent with the previously published data in terms of the distribution of blood groups.

However, the distribution of blood groups shows variability among the countries [20]. Group 0 is the most common blood type worldwide, especially in South and Central America. Group B is common in Asia, particularly in Northern India. Group A is common all over the world, although its highest incidence is seen among Australian Aborigines and Sami race. In England, the distribution of blood groups (A, B, 0, and AB) was reported in 45%, 9%, 43%, and 3%, respectively [20]. Again in Italy, the national distribution of blood types (A, B, 0, and AB) was reported by the Italian Blood Donors Association in 32.02%, 14.10%, 50.63%, and 32.24%, respectively [21]. Because blood groups differ depending on the geographic region, it is difficult to compare associations of blood groups with various diseases and medical conditions between the studies conducted in different countries. However, similar to our results, the majority of the abovementioned studies report a high incidence of blood group A in hip fractures [20, 21].

The relationship between AB0 groups and proximal femoral fracture was published for the first time by Buckwalter et al. in 1957 [22]. The authors compared 851 patients with fractures and 5517 healthy donors and found a higher incidence of fractures in group A. In a study by Thorsoe in 1960, no significant difference was found between the distribution of hip fractures between the patients and health controls, although the A blood group was reported to be more common in patients with intracapsular fractures [8]. In a recent study, Uzoigwe et al. reported that the A blood group was associated with intracapsular fractures in 2987 consecutive hip fractures [23]. Alffram found no significant difference between two fracture patterns in the A blood type [24]. Again, in a study by Toro et al. in Southern Italy, the AB0 system was not associated with proximal femoral fracture patterns [25]. Similarly, in our study, we could not find a significant correlation between blood types and fracture patterns of the patients (*p* = 0.205). In our study, the highest incidence of extracapsular fractures was found in the A blood group.

There are studies in the literature investigating the effects of some protocols on blood loss following femoral fracture surgery. Schiavone et al. reported that the use of tranexamic acid significantly decreased the amount of postsurgery blood loss and thus the need for transfusion in the surgical treatment of femoral fractures [26]. However, since our primary objective was to investigate the AB0 blood system, we did not evaluate the effects of such protocols. Accordingly, a significant difference was found between the amounts of transfusion according to blood types. Again, there was no significant difference between the blood groups in terms of the duration of hospitalization. There was no significant difference between the durations of hospitalization according to fracture patterns.

When patients were evaluated by age groups, the mean duration of hospitalization was significantly shorter in 46-55 and 56-65 years age groups compared to the >65 years age group. Similarly, in a study by Puckeridge et al., it was found that blood transfusion and hospitalization were higher in older hip fracture patients. They also found that the postoperative transfusion amount was higher in patients with extracapsular fractures [27]. No significant difference was found between the sexes in terms of the duration of hospitalization.

Studies in the literature have reported that not locking the nail distally reduces pain and bleeding and therefore the days of hospitalization. In the present study, we also have not locked the nails during the PFN procedure [28, 29]. When durations of hospitalization were compared according to the operation types, the mean duration of hospitalization was significantly shorter in cannulated screw and PFN operations than in the other types of surgical procedures.

One of the complications of proximal femoral fractures is nonunion and fractures around devices that bring the patient back to surgery [30]. However, since our primary objective was to investigate relationships between the AB0 blood system and hip fracture patterns, duration of hospitalization, and amount of blood transfusion, we did not investigate complications related to these fractures.

When patients were evaluated in terms of the amount of transfusion, no significant difference was found between the amounts of transfusion according to gender. There was a statistically significant difference between the amounts of transfusion according to the types of surgical procedures. The amount of transfusion was significantly higher in the PHA procedure compared to the PFN procedure.

To our knowledge, this study is the first study about the relationships between femoral fracture patterns and the AB0 blood system in Turkey. However, our study has some limitations. First, this study was designed as a retrospective evaluation and included a relatively small number of patients. Second, we used ICD codes to determine the fracture population. This might lead us to missing some data. More importantly, since there was no study in the literature investigating similar parameters with our study, we could not exactly compare our results.

## 5. Conclusion

There are various differences between intracapsular and extracapsular hip fracture patterns. The mechanisms causing these patterns are yet to be fully clarified. On the other hand, hereditary factors have also been implicated among these factors. It is proposed that the AB0 blood system could play a role in these patterns. However, in our study population, we could not find a significant relationship between the AB0 blood system and fracture patterns. Given the differences in the distribution of AB0 blood pressure among geographic regions, we believe that these potential relationships could be explained with further comprehensive studies with larger populations.

## Figures and Tables

**Table 1:** Fracture patterns and mean duration of hospitalization according to genders.

Gender	Patient number	Intracapsular	Extracapsular	Total
Male	134 (43.5%)	46 (44.7%)	88 (42.9%)	134
Female	174 (56.5%)	117 (57.1%)	117 (57.1%)	205
Total	308 (100%)	174 (43.5%)	174 (56.5%)	308

**Table 2:** Fracture patterns and mean duration of hospitalization according to age groups.

Age groups (years)	Patient number	Intracapsular	Extracapsular	Hospitalization (days)
20-45	13	7	6	2.92
46-55	17	12	5	3.8
56-65	35	12	23	3.6
66-75	50	16	34	5.1
>75	193	56	137	5.12

**Table 3:** Distribution of mean age, M/F ratio, and fracture patterns by AB0 blood groups.

Blood group	Mean age (years)	Male/female	Intracapsular	Extracapsular
A	75.85	0.78	48 (35.8%)	86 (64.2%)
B	75.91	0.72	27 (39.1%)	42 (60.9%)
0	75.25	0.75	21 (24.7%)	65 (75.6%)
AB	73.31	0.90	7 (36.8%)	12 (63.2%)

**Table 4:** Distribution of the duration of hospitalization and age group by surgical procedures.

Surgical procedure	Hospitalization (days)	20-45	46-55	56-65	66-75	>75
Cannulated hip screw (CHS)	2.08	4	4	3	0	1
Proximal femoral nail (PFN)	4.10	8	8	16	21	51
Partial hip arthroplasty (PHA)	5.20	0	0	6	16	135
Total hip arthroplasty (THA)	5.80	1	5	9	10	2
Dynamic hip screw (DHS)	4.0	0	0	1	0	3
Proximal femoral plate (PFP)	5.2	0	0	0	3	1

**Table 5:** Transfusion amounts by age groups, blood groups, and fracture patterns.

Transfusion amounts (units)	
Age groups (years)	
20-45	0.38
46-55	0.70
56-65	0.51
66-75	0.86
>75	0.86
Blood groups	
A	0.78
B	0.71
0	0.90
AB	0.63
Fracture patterns	
Intracapsular	0.72
Extracapsular	0.82

## Data Availability

Data used for this study are included in the article.
